# Remote Corticospinal Tract Degeneration After Cortical Stroke in Rats May Not Preclude Spontaneous Sensorimotor Recovery

**DOI:** 10.1177/15459683211041318

**Published:** 2021-09-21

**Authors:** Michel R. T. Sinke, Geralda A. F. van Tilborg, Anu E. Meerwaldt, Caroline L. van Heijningen, Annette van der Toorn, Milou Straathof, Fazle Rakib, Mohamed H. M. Ali, Khalid Al-Saad, Willem M. Otte, Rick M. Dijkhuizen

**Affiliations:** 1 Biomedical MR Imaging and Spectroscopy Group, Center for Image Sciences, University Medical Center Utrecht and Utrecht University, Utrecht, The Netherlands; 2 Department of Chemistry and Earth Sciences, 108740 College of Arts and Sciences, Qatar University, Doha, Qatar; 3 Neurological Disorders Research Center, Qatar Biomedical Research Institute (QBRI), 370593 Hamad Bin Khalifa University (HBKU), Doha, Qatar; 4 Department of Child Neurology, University Medical Center Utrecht and Utrecht University, 526115 UMC Utrecht Brain Center, Utrecht, The Netherlands

**Keywords:** brain, stroke, corticospinal tract, diffusion magnetic resonance imaging, diffusion tractography, behavior

## Abstract

*Background.* Recovery of motor function after stroke appears to be related to the integrity of axonal connections in the corticospinal tract (CST) and corpus callosum, which may both be affected after cortical stroke. *Objective.* In the present study, we aimed to elucidate the relationship of changes in measures of the CST and transcallosal tract integrity, with the interhemispheric functional connectivity and sensorimotor performance after experimental cortical stroke. *Methods.* We conducted in vivo diffusion magnetic resonance imaging (MRI), resting-state functional MRI, and behavior testing in twenty-five male Sprague Dawley rats recovering from unilateral photothrombotic stroke in the sensorimotor cortex. Twenty-three healthy rats served as controls. *Results.* A reduction in the number of reconstructed fibers, a lower fractional anisotropy, and higher radial diffusivity in the ipsilesional but intact CST, reflected remote white matter degeneration. In contrast, transcallosal tract integrity remained preserved. Functional connectivity between the ipsi- and contralesional forelimb regions of the primary somatosensory cortex significantly reduced at week 8 post-stroke. Comparably, usage of the stroke-affected forelimb was normal at week 28, following significant initial impairment between day 1 and week 8 post-stroke. *Conclusions.* Our study shows that post-stroke motor recovery is possible despite degeneration in the CST and may be supported by intact neuronal communication between hemispheres.

## Introduction

Stroke is one of the most prevalent neurological disorders worldwide and one of the main causes of adult disability, affecting almost 17 million people throughout the world.^1,2^ The majority of stroke survivors experience long-term sensorimotor and cognitive dysfunction. However, in the weeks and months following a stroke, many patients show partial recovery of sensorimotor (or cognitive) functions. In many cases, functional recovery obeys the recently postulated “proportional recovery rule,” which states that patients should reach about 70% of their maximum potential recovery at 3 months post-stroke.^3^ Cross-species validity of the proportional recovery rule has recently been demonstrated in a large study with rats recovering from ischemic stroke.^4^ However, the proportional recovery rule does not apply invariably, and many patients experience limited or no functional recovery over time.^3^ Furthermore, there has been debate in recent articles about the suitability of the proportional recovery rule from a statistical and methodological perspective.^5,6^ This strongly emphasizes the need for improved understanding of the underlying mechanisms of (spontaneous) functional recovery after stroke and the identification of biomarkers that can accurately predict functional outcome.

Post-stroke functional recovery has been related to reorganization and restoration of surviving ipsi- and contralesional networks in the brain.^7-11^ A critical factor for functionally effective neuronal reorganization within the motor system appears to be the integrity of axonal connections in the corticospinal tract (CST) and corpus callosum. However, after stroke, these pathways may be structurally or functionally disrupted, thereby directly affecting motor function along with potential for motor recovery in stroke survivors.^7,12-15^

Structural and functional connectivity can be mapped at a whole brain level with *in vivo* magnetic resonance imaging (MRI) techniques, such as diffusion MRI and functional MRI. Diffusion MRI indirectly probes the arrangement of axonal projections from the random diffusion of water molecules in and around axonal fiber bundles.^16,17^ Diffusion MRI studies in stroke patients have revealed reduced fractional anisotropy (FA) and increased diffusivity values in the ipsilesional CST, indicative of axonal degeneration. Higher FA and lower diffusivity values are associated with improved motor functions at chronic stages.^18-21^ Also, measures of the intactness of transcallosal tracts have been shown to be predictive of good motor outcome.^19,22^

Functional connectivity within brain networks may be assessed with resting-state functional MRI (rs-fMRI), which measures inter-regional temporal correlation of low-frequency spontaneous fluctuations in blood oxygenation level–dependent signals, reflective of neuronal synchronization. Clinical and pre-clinical studies have reported post-stroke loss of functional connectivity, for example, between the left and right motor cortices, which may recover over time, in parallel with recovery of motor function.^7,15,22-25^ These findings also point toward the importance of transcallosal tracts—and their integrity—for functional recovery.

Despite increasing insights into the importance of CST and transcallosal tracts for motor recovery after stroke, the relationships between stroke injury, motor function, and structural and functional connectivities within the remaining post-stroke motor network remain unknown. In the present study, we aimed to elucidate the relationship of changes in measures of CST and transcallosal tract integrity vs cortical injury, functional connectivity, and motor performance chronically after experimental cortical stroke. To that aim, we acquired in vivo diffusion MRI, and rs-fMRI data, and behavioral scores from rats at week 1, 8, or 28 after unilateral focal stroke in the motor cortex.

## Methods

### Animals

Ethical approval was given by the Animal Experiments Committee of the University Medical Center Utrecht and the Utrecht University, and experiments were performed in accordance with the guidelines of the European Communities Council Directive.

The present study was performed with 48 adult male Sprague Dawley rats (Charles River, Germany). An overview of animal weights and ages is given in Table 1. Animals were allowed to acclimatize at least 7 days after delivery. Animals were housed under standard conditions with a light/dark cycle of 12/12 hours, with light on time at 7:00 a.m, and *ad libitum* access to food and water. A power calculation was conducted with *Gpower 3.1.5.* A sample size (n = 7) per time point was computed based on a t-test for the difference between independent means, with α = .05, power = 80%, and an effect size of 1.5. The effect size was based on FA values in the anterior internal capsule of rats 7 days post-stroke vs control rats without stroke from a previous study.^22^ Hence, a total of 25 animals were subjected to experimental stroke surgery. Three animals died as a consequence of stroke surgery before MRI acquisition. MRI was performed in control animals and animals with stroke at 1 week (n = 7 control, n = 8 stroke), 8 weeks (n = 8 control, n = 7 stroke), or 28 weeks (n = 8 control, n = 7 stroke). In addition, each rat underwent behavioral testing (during light-on time) at multiple time points, up to the day of MRI.

**Table 1.** Body Weights and Ages of Rats in the Experimental Groups.

Time point	Body weight at day of stroke onset (g, mean ± SD)	Body weight at day of MRI (g, mean ± SD)		Age at day of MRI (weeks)
	*Stroke*	*Stroke*	*Control*	*Stroke and Control*
*Week 1*	423 ± 16	450 ± 19	444 ± 8	12
*Week 8*	425 ± 19	555 ± 19	580 ± 54	19
*Week 28*	426 ± 21	787 ± 49	730 ± 89	39

Abbreviations: MRI, magnetic resonance imaging.

### Stroke Model

Photothrombotic stroke was induced in the right sensorimotor cortex of adult male Sprague Dawley rats at 11 weeks of age (n = 25, for body weights see Table 1), as described in the Supplemental Material. Surgery was performed under isoflurane anesthesia supplied in a mixture of O_2_/air (1/4) (4% for induction; 1.5–2.0% for maintenance). Rats were mechanically ventilated (UNO Micro Ventilator-03, UNO b.v., The Netherlands) through an endotracheal tube (Abbocath^TM^-T, 14Gx51 mm, ICU Medical, USA). Blood oxygenation and heart rate were continuously monitored (Nonin Medical 8500V pulse oximeter) and body temperature was maintained at 37 ± .5°C.

### MRI Acquisition

MRI was performed on a 9.4T horizontal bore Varian MR System (Palo Alto, CA, USA), equipped with a gradient insert with an inner diameter of 12 cm and a maximum gradient strength of 400 mT/m at a rise time of 130 µs. A home-built Helmholtz volume coil (90 mm diameter) and an actively decoupled surface coil (25 mm diameter) were used for excitation and reception, respectively.

For MRI, rats were anesthetized (4% isoflurane induction; 1.5–2.0% isoflurane maintenance in O_2_/air (1/4)), endotracheally intubated (Abbocath^TM^-T, 14G × 51 mm, ICU Medical) and mechanically ventilated (TOPO Small Animal Ventilator Kent Scientific). Oxygen saturation, heart rate (Nonin Medical 8600V pulse oximeter), and expired CO_2_ (Microstream handheld capnograph, Oridion) were continuously measured and kept within physiological range. Temperature was maintained at 37.0 ± .5°C. To minimize motion artifacts, animals’ heads were fixed with ear- and toothbars inside a home-built animal cradle. Rat heads were carefully positioned underneath the surface coil to obtain full coverage of the cerebrum.

Voxel-based shimming was performed on a rectangular voxel covering the cerebrum and a small fraction of the olfactory bulb and cerebellum, using gradient-echo 3D shimming. Directly following shimming, inspired isoflurane levels were reduced to 1.5% and the imaging protocol was started.

The imaging protocol consisted of anatomical imaging, rs-fMRI, and diffusion MRI. Anatomical images were acquired using a balanced steady-state free precession sequence with an isotropic spatial resolution of 250 μm (see Supplemental Material for parameter settings).

Resting-state fMRI (rs-fMRI) was performed using a single-shot 3D gradient-echo EPI sequence with an isotropic spatial resolution of 600 μm (see Supplemental Material for parameter settings).

Right after rs-fMRI, the inspired isoflurane level was increased to 2% and maintained until the end of the MRI protocol. Diffusion MRI was executed with a 4-shot 2D spin-echo EPI sequence with diffusion-weighting (see Supplemental Material for parameter settings). For each acquisition, 2 equivalent b_0_ images without diffusion-weighting were also obtained.

### MRI Data Processing

Acquired MR images were post-processed with FSL 5.0 software.^26^

### Anatomical Imaging–Lesion Segmentation

The brain voxels in the anatomical images were extracted.^27^ Images were corrected for signal inhomogeneities and registered to the brain scan of one reference rat from the 1 week stroke group, with affine transformation (FLIRT)^28,29^ followed by non-linear transformation (FNIRT).^30^ Brain lesions were manually segmented in the aligned anatomical images using FSLview. Subsequently, a lesion incidence map was calculated.

### Diffusion Tensor Imaging and Tractography

Brain voxels in the diffusion scans were extracted, based on the b_0_ images.^27^ Next, FSL’s DTIFIT was used to calculate FA, mean diffusivity (MD), axial diffusivity (AD), and radial diffusivity (RD) from the masked diffusion data. Images from 2 control animals at 1 week were excluded from further analyses because of poor image quality. Deterministic diffusion tensor-based whole-brain tractography was performed in MRtrix3,^31^ generating 250.000 streamlines with a step size of 25 μm, an FA threshold ≥ .3 and an angle threshold of 30° (Supplemental Figure 1). Procedures for image registration are described in the Supplemental Material.

To select the left and right CST from the 250.000 streamlines, inverse transformations were applied to register the bilateral internal capsule and striatum (consisting of caudate putamen and globus pallidus atlas regions) back from the reference space to individual animal space.^28,29^ Additionally, bilateral brain stem (approximately −7 mm posterior from bregma) and optic fiber (approximately −1.5 mm from bregma) regions were manually outlined for each individual animal, which were used to include or exclude streamlines, respectively. The internal capsule also served to include streamlines in the CST selection process. Only streamlines that passed the brain stem and internal capsule were included. In many rats, some streamlines deviated and bent away from the main bundle towards the optic tract. These streamlines were excluded using a manually drawn exclusion area. Next, parts of streamlines that went through the striatum were excluded (i.e., masked out) for further analysis. As a last step, spurious “sprouting” streamlines were manually removed from the CST segmentations (Supplemental Figure 1).

**Figure 1.**
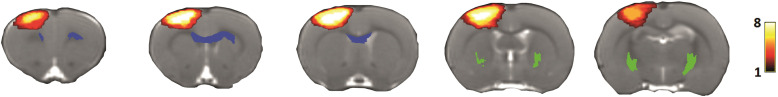
Lesion incidence map and segmented tracts overlaid on anatomical rat brain images at one week post-stroke. Anatomical rat brain images of coronal slices from anterior (*left*) to posterior (*right*). Lesion incidence is scaled from 1 (*dark red*) to 8 (*yellow*), indicating the number of rats with a lesion in that particular region. The bilateral CST (*green*) and transcallosal tracts (*blue*) reconstructions are also shown. CST, corticospinal tract.

To select the anterior transcallosal tracts, which contain interhemispheric connections between sensorimotor regions, we manually extended the bilateral primary motor (M1) regions in the reference space (ventral direction only), resulting in M1 regions that include underlying white matter. Inverse transformations were applied to register the extended bilateral M1 regions and corpus callosum back from the reference space into individual animal space using FLIRT and FNIRT.^28,29^ We selected all streamlines that passed through the bilateral extended M1 regions and the corpus callosum. Streamlines were cut off at the outside borders of the bilateral M1 regions. The position of the resulting CST and transcallosal tracts are shown as overlays on the lesion incidence map in Figure 1.

Along-tract analysis was performed to obtain the average FA, MD, AD, and RD along left and right CSTs as well as the transcallosal tract. In addition, we also determined the number of streamlines for the left and right CST, and the transcallosal tract. One DTI dataset of a control rat at 1 week could not be included as it was not saved due to a scanner-related problem. Another DTI dataset of a control rat at 1 week had to be excluded because of bad image quality. Resulting group sizes were n_control_ = 6, n_stroke_ = 8 (at 1 week), n_control_ = 8, and n_stroke_ = 7 (at 8 weeks) and n_control_ = 8 and n_stroke_ = 7 (at 28 weeks).

### Resting-State Functional Connectivity

The first 20 images of the rs-fMRI time-series were discarded to ascertain steady-state. Next, motion correction was performed to the mean image using FSL’s MCFLIRT, and motion correction parameters were used for regression of the motion-corrected images using FSL’s fsl_glm. The time-series was then bandpass-filtered between .01 and .1 Hz using AFNI’s 3dFourier tool.

Rs-fMRI images were first registered to a reference rat brain (see Supplemental Material), followed by registration to the Paxinos and Watson rat brain atlas,^32,33^ from which we selected 16 regions of interest (ROIs), that is, the bilateral caudate putamen, M1, secondary motor cortex (M2), forelimb and hindlimb regions of the primary somatosensory cortex (S1FL and S1HL, respectively), secondary somatosensory cortex, thalamus, and medial prefrontal cortex (mPFC). A temporal-signal-to-noise (tSNR) mask was obtained from the masked motion-corrected rs-fMRI time-series for each rat, by calculating tSNR images and subsequent thresholding of these images at values ≥ 10. The resulting tSNR mask was applied to the selected ROIs, and seed-based Fisher’s z-transformed functional connectivity maps and matrices were calculated from the motion-corrected and regressed rs-fMRI time-series for each rat. Group-average tSNR maps are shown in Supplemental Figure 2.

Seed-based functional connectivity maps with contralesional forelimb or hindlimb regions of the primary somatosensory cortex as seed regions were used to assess data quality. Four datasets were excluded because of major artifacts in the functional connectivity maps (one stroke animal at week 1 and three control animals at week 8). One additional dataset could not be included as it was not saved due to a scanner-related problem (control week 1). Resulting group sizes were n_control_ = 6 and n_stroke_ = 7 (at 1 week), n_control_ = 5 and n_stroke_ = 7 (at 8 weeks), and n_control_ = 8 and n_stroke_ = 7 (at 28 weeks).

### Sensorimotor Function Test

Animals were placed in a transparent Perspex vertical cylinder (20 cm diameter, 30 cm height), which was positioned on a horizontal elevated transparent Perspex plate. Videos of the animal were acquired with a video camera (Panasonic HC V520) located underneath the cylinder. We aimed to record 20 paw placements at minimum. The video analysis procedure is described in the Supplemental Material.

For each animal, the first 20 paw placement scores were included for calculation of the forelimb-use score. Subsequently, sum scores were calculated for the paw placements, resulting in a total paw placement score for “left” (PP_left_), “right” (PP_right_), and “both” (PP_both_). Forelimb use, expressed as the percentage usage of the left (impaired) forelimb (LF), was calculated according to the following equation

$$LF=\;\left(\left(\frac{\left(PP_{\text{left }}+PP_{\text{both}}/2\right)}{\left(PP_{\text{left}}+PP_{\text{right}}+PP_{\text{both}}\right)}\right)\right)\cdot 100\%$$

### Statistical Analyses

All statistical analyses and visualization were performed in *R 3.2* (http://www.r-project.org/), using the packages *ggplot2, plyr*, and *reshape2.* We used two-way ANOVA to test changes over time and differences between groups (i.e., stroke vs controls) in behavior, diffusion parameters, and functional connectivity. Post-hoc analysis was performed using the post-hoc Tukey test. We used Pearson’s correlation coefficient to determine the relationship between diffusion parameters, interhemispheric functional connectivity and behavior at the day of MRI. All statistical tests were corrected for multiple comparisons using the false discovery rate (FDR) method. Results with a FDR-corrected *P*-value < .05 were regarded as significant. For correlation analyses with behavioral measurements, we used the average of post-stroke day 1 and week 1 data as first post-stroke behavioral performance score.

## Results

### Brain Lesion

Unilateral cortical lesions were evident on anatomical MRI scans at 1 week post-stroke. Lesion incidence maps are depicted in Figure 1 and Supplemental Figure 3, showing the limited inter-subject variance of the lesion location. The mean (±SD) lesion volumes were 34.9 ± 10.9 mm^3^, 13.8 ± 3.8 mm^3^, and 6.0 ± 2.6 mm^3^ at 1, 8, and 28 weeks post-stroke, respectively. Gradual reduction of photothrombotically induced lesion volume on MRI has been reported before and can be explained by edema resorption and cellular infiltration.^34^ The lesion covered the sensorimotor cortex, with no or minimal involvement of underlying white matter. 46 ± 15% of M1, 22 ± 15% of M2, 63 ± 15% of S1FL, and 69 ± 21% of S1HL (mean ± SD) were affected by the lesion at 1 week after stroke.

### Corticospinal Tract and Transcallosal Tract Integrity

Reconstructions of the segmented transcallosal tracts and CST, displayed in Supplemental Figures 1 and 4, showed that these tracts were not part of the ischemic lesion.

**Figure 2.**
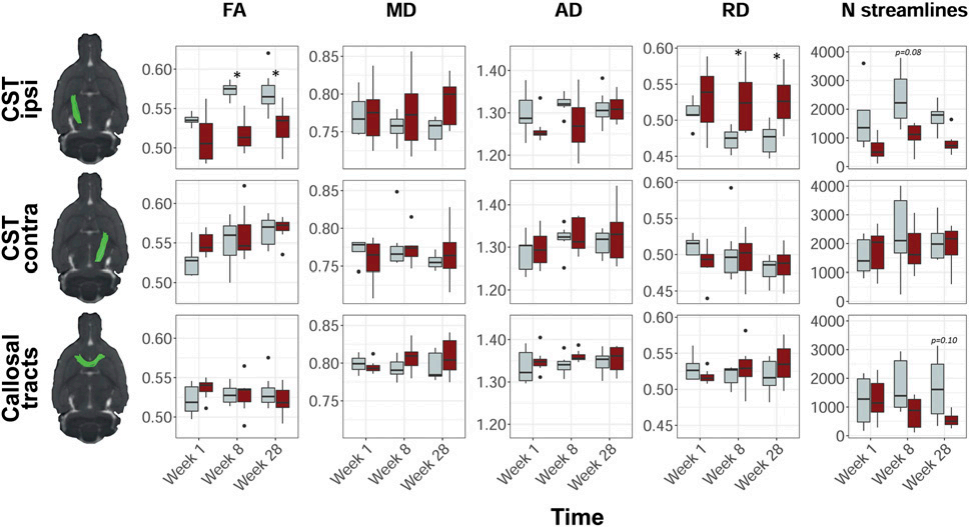
Diffusion MRI-based white matter characteristics in stroke and control rats. Representative segmentations of the ipsilesional CST (*top row*), contralesional CST (*middle row*), and transcallosal tracts (*bottom row*) of a control rat projected on an anatomical rat brain image (sagittal plane). Average fractional anisotropy (FA), mean diffusivity (MD), axial diffusivity (AD), radial diffusivity (RD), and number of streamlines, in control (*gray*) and stroke (*red*) rats, at 1 week, 8 weeks, and 28 weeks post-stroke are shown for each reconstructed white matter pathway. Boxplots show the median and inter-quartile range (IQR), whiskers represent 1.5 times the IQR, and dots represent outliers. MD, AD, and RD values are scaled ( × 10^−3^ mm^2^/s). *P < .05 (stroke vs control). CST, corticospinal tract; FA, fractional anisotropy; MD, mean diffusivity; AD, axial diffusivity; RD, radial diffusivity; IQR, inter-quartile range.

Figure 2 shows representative segmentations (in controls) of the bilateral CST and transcallosal tracts, their diffusion MRI-based characteristics, and their number of streamlines. We found a significantly lower average FA along streamlines in the ipsilesional CST of stroke rats as compared to control rats (Δ_FA_ = −.044, 95% CI = −.058 to −.030, *F*_*group*_ = 39.71, *P* < .0001). Post-hoc tests showed significant differences between FA over the ipsilesional streamlines in stroke and controls at 8 weeks (Δ_FA_ = −.056, 95% CI = −.092 to −.021, *P* < .001) and 28 weeks post-stroke (Δ_FA_ = −.042, 95% CI = −.078 to −.010, *P* < .05). In addition, the average RD along streamlines in the ipsilesional CST of stroke rats was significantly higher than in controls (Δ_RD_ (10^−3^ mm^2^/s) = .045, 95% CI = .025 to .065, *F*_*group*_ = 20.32, *P* < .0001). Post-hoc tests showed significant differences in RD over the ipsilesional streamlines at 8 weeks (Δ_RD_ (10^−3^ mm^2^/s) = .052, 95% CI = .001 to .103, *P* < .05) and 28 weeks post-stroke (Δ_RD_ (10^−3^ mm^2^/s) = .053, 95% CI = .002 to .104, *P* < .05). Although not statistically significant, AD over the ipsilesional CST streamlines tended to be lower in stroke rats at 1 week and 8 weeks as compared to controls. MD over the ipsilesional CST was not significantly different between stroke and controls rats. For the contralesional CST and the transcallosal tracts, we did not find significant differences over time, or between stroke and control rats.

In stroke rats, the number of CST streamlines in the ipsilesional (right) hemisphere was significantly lower than the number of CST streamlines in the contralesional (left) hemisphere (ΔCST_streamlines_ = −556, 95% CI = −888 to −225, *F*_*side*_ = 11.20, *P* < .01), and the number of CST streamlines in the left and right CST of control rats (ΔCST_streamlines_ = −670, 95% CI = −1002 to −339, *F*_*group*_ = 16.27, *P* < .0001). Post-hoc testing showed a trend toward a lower number of ipsilesional streamlines after stroke as compared to controls at 8 weeks (ΔCST_streamlines_ = −1280, 95% CI = −2626 to 67, *P* = .08). The number of streamlines in the corpus callosum (CC) was significantly lower after stroke as compared to control rats (ΔCC_streamlines_ = −684, 95% CI = −1163 to −206, *P* < .01), which tended to be most apparent at 28 weeks after stroke (ΔCC_streamlines_ = −1082, 95% CI = −2285 to 120, *P* = .10). Supplemental Table 1 shows the number of streamlines per time point, ROI and group.

### Resting-State Functional Connectivity

**Figure 3.**
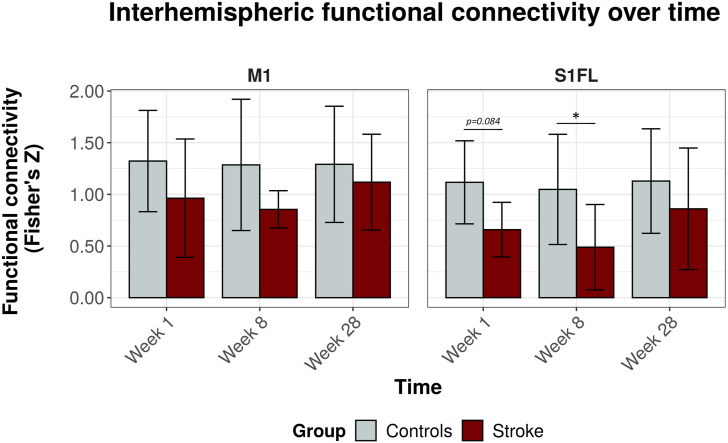
Interhemispheric functional connectivity over time for M1 and S1FL. Functional connectivity as mean (± SD) Fisher’s z for M1 (*left*) and S1FL (*right*) at 1 week, 8 weeks, and 28 weeks post-stroke (*x-axis*), for both controls (*gray*) and stroke rats (*red*). *P < .05 (stroke vs control). *M1 =* primary motor cortex*; S1FL =* forelimb regions of the primary somatosensory cortex*.*

Figure 3 shows the interhemispheric functional connectivities for the bilateral S1FL and the bilateral M1. Interhemispheric functional connectivity of S1FL was significantly lowered in stroke rats as compared to control rats (*F*_*group*_ = 8.8, *P* = .005). A similar trend was found for interhemispheric connectivity of M1 (*F*_*group*_ = 4.1, *P* = .050). Post-hoc tests revealed significant differences in interhemispheric functional connectivity of S1FL between stroke and control rats (ΔFC_S1FL_) at week 8 (ΔFC_S1FL_ = .56, 95% CI = .01 to 1.11, *P* = .046), and a similar trend at week 1 (ΔFC_S1FL_ = .46, 95% C.I. = −.06 to .98, *P* = .084). At 28 weeks, there was no significant difference in S1FL’s interhemispheric functional connectivity between stroke and control rats (ΔFC_S1FL_ = .27, 95% CI = −.22 to .76, *P* = .269). Interhemispheric functional connectivity values for the other sensorimotor ROIs are shown in Supplemental Figure 5. Of these ROIs, only S1HL had a significantly lower interhemispheric functional connectivity after stroke as compared to control rats (*F*_*group*_ = 9.9, *P* = .003). Interhemispheric functional connectivity values between contralesional sensorimotor ROIs and an ipsilesional reference region in the default mode network, that is, mPFC, did not show significant differences between stroke and control rats (Supplemental Figure 6).

### Functional Deficits

**Figure 4.**
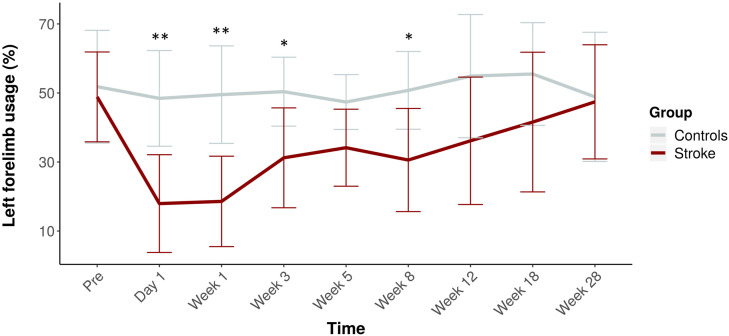
Usage of the left, that is, stroke-affected, forelimb over time. Mean (± SD) forelimb usage before stroke and at 1 day, and 1, 3, 5, 8, 12, 18, and 28 weeks post-stroke (*red*). The time-course for controls is shown in gray. **P < .01 and *P < .05 (stroke vs control).

Figure 4 shows the cylinder test results over time for stroke and control rats. We found a significant decline in left (stroke-affected) forelimb usage between stroke and control groups (LF (%) = −16.2, 95% CI = −12.4 to −20.1, *F*_*group*_ = 70.3, *P* < .0001). Control rats used their left and right forelimbs about equally over all time points. However, stroke rats showed significantly reduced use of the stroke-affected, that is, left, forelimb from the acute stages until 8 weeks post-stroke (*F*_group*time_ = 3.9, *P* < .001), which normalized during the chronic stages (*F*_*time*_ = 5.4, *P* < .0001).

Pearson’s correlation coefficients revealed no significant correlations between stroke-affected forelimb usage and diffusion parameters along the ipsilesional CST and CC streamlines or interhemispheric functional connectivity of the sensorimotor ROIs.

## Discussion

In the present study, we combined diffusion MRI, rs-fMRI, and behavioral testing to determine to what extent corticospinal and transcallosal tracts are affected in rats recovering from focal cortical stroke and how this may be linked to changes in functional network connectivity and sensorimotor performance. Diffusion MRI revealed a reduction in the number of streamlines, lower FA values, and higher RD values in the ipsilesional CST outside the cortical lesion area. These signs of remote white matter degeneration increased between 1 and 28 weeks post-stroke. The (time-course of) structural features of the transcallosal tracts did not significantly differ between recovering stroke animals and control animals. Resting-state fMRI showed loss of interhemispheric functional connectivity between homologous ipsi- and contralesional S1FL at 1 and 8 weeks after stroke, which was partially restored after 28 weeks. Correspondingly,, use of the stroke-affected forelimb was significantly impaired during the first 8 weeks after stroke, but gradually recovered towards week 28.

To map structural white matter tracts, we applied an advanced approach of diffusion MRI-based tractography that has been effectively utilized in stroke patient studies.^19,21,35^ Recently, this technique has also been successfully applied and histologically validated for reconstruction of different white matter tracts in rats,^36^ thereby offering a powerful and unique means for *in vivo* assessment of the large white matter tracts in rodents at a whole-brain level. In the present study, we extended this method by developing a pipeline for mapping, segmenting, and analyzing the bilateral CST from the rat brain.

So far, the development of changes in the integrity of the CST has hardly been investigated in pre-clinical stroke studies despite its known significance in (loss and recovery of) sensorimotor function. The diffusion MRI-based tractography approach in the present study provided different metrics indicative of CST integrity as well as the number of reconstructed streamlines. In contrast to metrics such as FA, MD, RD, and AD, the number of streamlines is not a quantitative measure of white matter density or integrity. The number of reconstructed streamlines was found to be highly variable across rats and time points. This may be partly attributed to the lack of normalization procedures, such as between-subject intensity normalization or tractogram filtering procedures like spherical-deconvolution informed filtering of tractograms (SIFT).^37,38^ However, our rat brain data were not well suited for these procedures that have been developed for human brain data with higher numbers of diffusion-weighting directions and voxels with less anisotropic dimensions. In the present study, we tried to minimize within- and between-subject variation by generating a large number of streamlines (250.000) across the entire brain to ensure the reconstruction of specific tracts such as the corpus callosum and CST.

The reduced number of streamlines that we found in the ipsilesional CST after ischemic cortical damage in rats is in line with recent diffusion MRI studies in human stroke patients reporting on reduced structural connectivity (strength) in the ipsilesional CST.^21,39,40^ Furthermore, across all time points, we measured reduced FA in the ipsilesional CST of stroke rats as compared to controls. This has also been described in earlier stroke studies in rats and human patients^18-22,41^ and is believed to reflect anterograde (Wallerian) degeneration or extensive white matter edema. An increase in RD, also evident in our data, has previously been related to demyelination of white matter tracts.^41-43^ Because the segmentations of the CST were located entirely outside the lesioned territory, the observed structural changes are believed to be due to remote degeneration of fiber tracts that are structurally connected to the infarcted cortex. Noteworthy, in the CST of control rats, FA tended to increase over time, that is, between 12 and 39 weeks of age. This has previously been associated with ongoing white matter maturation in rats from early to late adulthood.^44,45^ As such, the larger difference in FA along the ipsilesional CST between control rats and stroke rats in the chronic phase may also be indicative of disturbed white matter maturation.

Despite the indication of remotely degenerating corticospinal fibers after cortical ischemic injury, our study did not reveal similar signs of degeneration of transcallosal tracts, which are known to also arise from the sensorimotor cortex. Although speculative, we think that recruitment of intact contralesional homotopical cortical regions, which may play a compensatory role in sensorimotor recovery^15,22^ and sustained interhemispheric neuronal communication through the transcallosal tracts, may counteract or limit secondary white matter degeneration in these tracts. In contrast, decreased usage of the contralateral forelimb, and consequent reduced axonal in- and output may enhance the neurodegenerative processes of the ipsilesional CST (i.e., “use it or lose it”).^46,47^ Preservation of transcallosal tracts may also have contributed to the preservation of functional connectivity between most of the intact ipsi- and contralesional regions within the sensorimotor network after stroke, as well as to the restoration of interhemispheric functional connectivity of S1FL at 28 weeks. This is in agreement with an earlier study, in which we showed restoration of interhemispheric functional connectivity at 10 weeks after unilateral transient middle cerebral artery occlusion in rats, following initial loss of function during the first 3 weeks, together with intact trans-hemispheric neuroanatomical connectivity.^15^ Moreover, improved sensorimotor performance was related to normalization of interhemispheric functional connectivity,^15^ a finding that has also been reported in human stroke studies.^23,48^

Our current study shows that despite remote CST degeneration, recovery of sensorimotor function is possible, which may rely on the integrity of transcallosal tracts, although our data do not provide evidence of causality between microstructural integrity and functional connectivity. Other mechanisms, like cortical remapping^49-52^ or increased involvement of other white matter tracts, such as the rubro-spinal tract^39,53,54^ and reticulospinal tract,^55,56^ may also contribute to alleviation of functional impairments. Future research could employ recently developed quantitative diffusion MRI-based approaches, which enable calculation of fiber density and fiber cross-section,^57-60^ for improved assessment of white matter characteristics in relation to (potential for) stroke recovery. This may lead to the development of novel diagnostic imaging markers that could better predict functional outcome in patients recovering from stroke and guide individualized treatment strategies.

## Supplemental Material

sj-pdf-1-nnr-10.1177_15459683211041318 – Supplemental Material for Remote Corticospinal Tract Degeneration After Cortical Stroke in Rats May Not Preclude Spontaneous Sensorimotor Recovery Click here for additional data file. Supplemental Material, sj-pdf-1-nnr-10.1177_15459683211041318 for Remote Corticospinal Tract Degeneration After Cortical Stroke in Rats May Not Preclude Spontaneous Sensorimotor Recovery by Michel R. T. Sinke, Geralda A. F. van Tilborg, Anu E. Meerwaldt, Caroline L. van Heijningen, Annette van der Toorn, Milou Straathof, Fazle Rakib, Mohamed H. M. Ali, Khalid Al-Saad, Willem M. Otte and Rick M. Dijkhuizen in Neurorehabilitation and Neural Repair
