# Supplementary material for: Remote Corticospinal Tract Degeneration After Cortical Stroke in Rats May Not Preclude Spontaneous Sensorimotor Recovery
Source: Neurorehabil Neural Repair. 2021 Sep 21;35(11):1010–9. doi: 10.1177/15459683211041318 (PMC8593321; doi:10.1177/15459683211041318)
Supplement: sj-pdf-1-nnr-10.1177_15459683211041318 – Supplemental Material for Remote Corticospinal Tract Degeneration After Cortical Stroke in Rats May Not Preclude Spontaneous Sensorimotor Recovery [file sj-pdf-1-nnr-10.1177_15459683211041318.pdf]

Supplementary Material

Methods

Photothrombotic stroke induction

Rats were placed in a stereotaxic frame. The scalp was opened, and lidocaine (Xylocaine 100 mg/ml, AstraZeneca BV, Zoetermeer, The Netherlands) was sprayed on the periosteum, after which the periosteum was removed and the skull was allowed to dry. Next, the illumination area was delineated between +4 to -4 mm anterior/posterior and 1.5 to 4.5 mm lateral to bregma, using black tape. Subsequently, an optic fiber (diameter 4.5mm), mounted on a cold light source (Schott KL 1500 LCD, Germany), with a green fluorescent filter (wavelength 515 nm), was placed on the skull. The light source was used at a color temperature of 3200K (setting 5), and aperture setting E. Prior to illumination, the photosensitive dye Rose Bengal (Sigma) was infused through the vena saphena (25 mg/kg) at an infusion rate of 5.625 mg/min in a darkened room. Directly after infusion, illumination was started, which lasted for 20 minutes. Following illumination, the skin on the leg and head was sutured, and the animals were placed on a heating pad in order to recover. Once awake, animals received one single dose (0.05 mg/kg) of buprenorphine (Temgesic, Schering-Plough).

MRI sequence parameter settings

Anatomical balanced steady-state free precession sequence: repetition time (TR) = 5 ms, echo time (TE) = 2.5 ms, flip angle = 20°, field of view (FOV) = 40 × 32 × 24 mm^3, matrix = 160 × 128 × 96, 3 averages and pulse angle shifts of 0°, 90°, 180° and 270°, total scan time = 12 min.

Resting-state single-shot 3D gradient-echo EPI sequence: TR = 730.8 ms, TE = 15 ms, flip angle = 13°, bandwidth = 250 kHz, FOV = 32.4 × 32.4 x 16.8 mm^3, matrix = 54 × 54 × 28, transverse orientation, 800 images, total scan time ≈ 9.5 min.

Diffusion-weighted 4-shot 2D spin-echo EPI sequence: TR = 1.7 s, TE = 34 ms, bandwidth = 250 kHz, FOV = 32 × 32 mm^2, matrix = 64 × 128, 25 transverse slices of 0.5 mm thickness, 4 averages, 30 gradient directions, diffusion gradient strength = 19 G/cm, Δ = 15.5 µs, δ = 6.5 µs, b = 1471 s/mm^2, total scan time = 14.5 min.

Image processing for tractography

Prior to selection of the corticospinal and transcallosal tracts, image registration was performed according to the following procedure. After signal inhomogeneity correction, the b_0 brain images for each individual rat were registered to the b_0 image of one reference rat brain (8 weeks, control group) with linear[1,2] and non-linear transformations.[3] The mean b_0 image of all control rat brains was subsequently registered linearly and nonlinearly to a 3D reconstruction of the Paxinos and Watson rat brain atlas[4,5], with some manual refinement to the transformed internal capsule and corpus callosum.

Registration of rs-fMRI data

For registration purposes, a mean image of the motion-corrected rs-fMRI time-series was calculated. The resulting mean rs-fMRI image for each rat was corrected for homogeneity and linearly registered to the mean rs-fMRI image of a reference rat brain (control group, 8 weeks). Registered images from all rats were used to calculate a mean rs-fMRI image in reference space, which was used to obtain a brain mask in reference space. The resulting brain mask was inversely transformed to subject space, and applied to the mean rs-fMRI image for each rat in subject space.

Paw placement analysis

Videos were analyzed with BORIS software, by an observer blinded to the experimental group and time point. Paw placement was scored as described by Hua et al., 2002[6]. Each time the animal reared from a sitting position, or when starting in upright position without touching the cylinder wall, subsequent placement of the entire forelimb(s) against the cylinder wall were scored as paw placement ‘left’, ‘right’ or ‘both’ for independent or simultaneous use of the left and right forelimb, respectively. Touching the cylinder wall with digits only was not scored as paw placement. When the rat was in upright position, each time one paw was horizontally repositioned on the cylinder wall, directly followed by repositioning of the second paw on the cylinder wall, placement was scored as ‘both’. In case the second paw was not repositioned or removed from the cylinder wall it was not scored as paw placement.

Figures

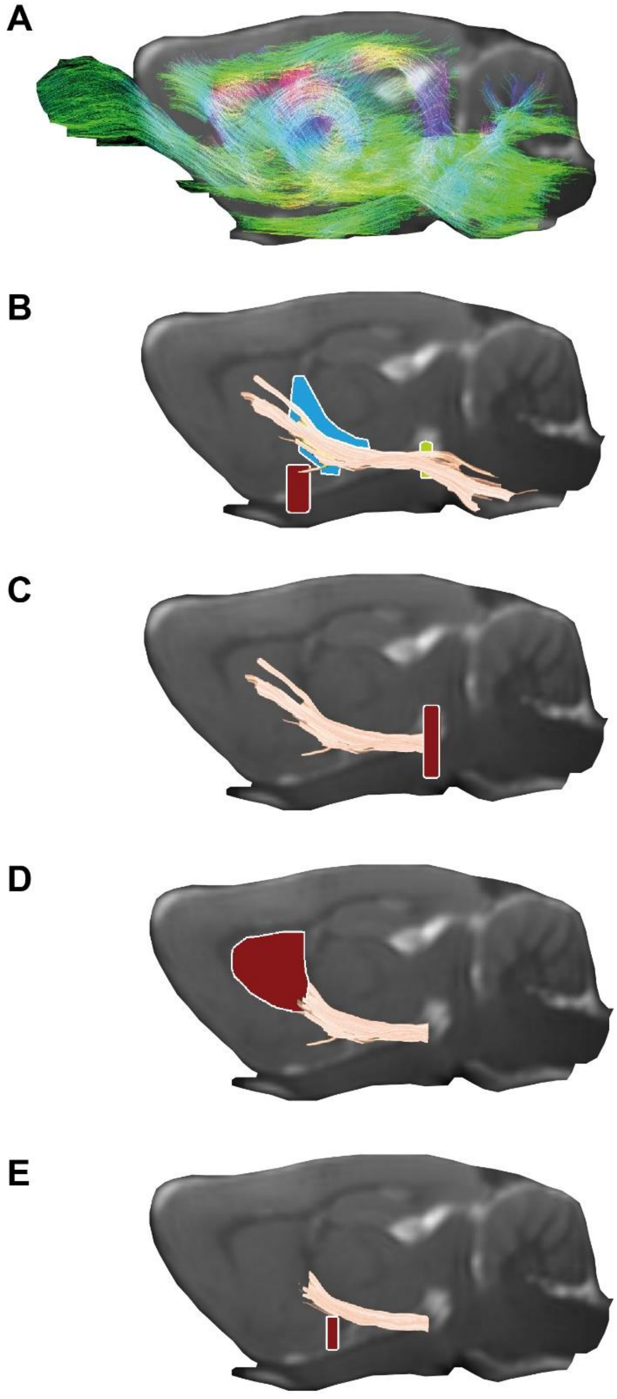

Suppl. Figure 1. Schematic representation of the CST segmentation pipeline. (A) Whole-brain tractogram projected on an anatomical image of the rat brain (sagittal plane). The colors indicate the direction of the streamlines (blue = inferior-superior; red = medial-lateral; green = anterior-posterior). **(B)** From the whole-brain tractogram all streamlines were selected that

passed through the brainstem (*green rectangle*) and internal capsule (*blue region*), but not the optic tract (*red rectangle*). **(C)** Subsequently, streamlines were cut off at the level of the brainstem (*red rectangle*). **(D)** Parts of streamlines that entered the striatum (*red region*) were masked out. **(E)** Finally, spurious tracts were removed through manually drawn masks (*red rectangle*).

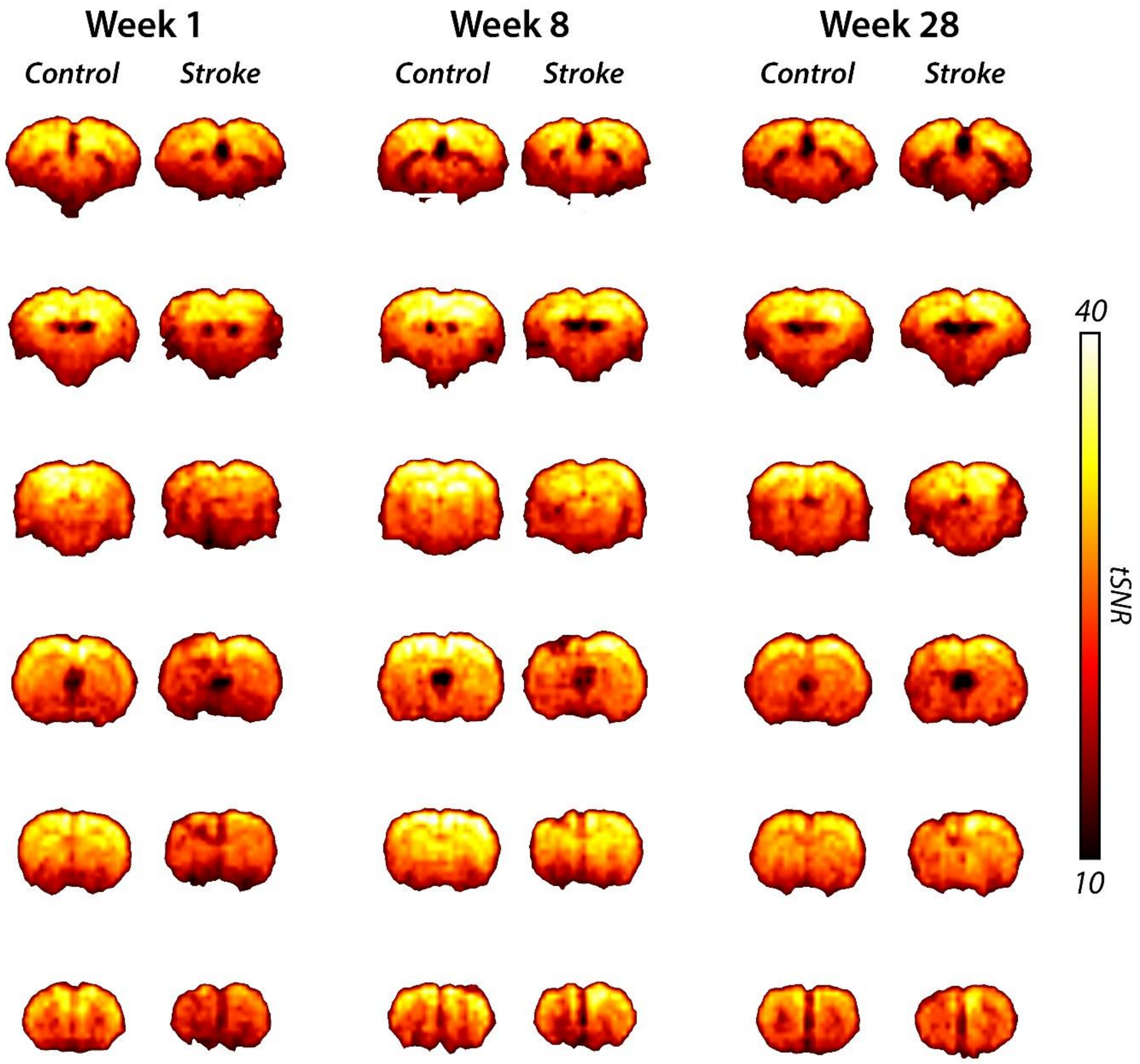

Suppl. Figure 2. Group-average tSNR maps at 1, 8 and 28 weeks post-stroke, for control and stroke animals.

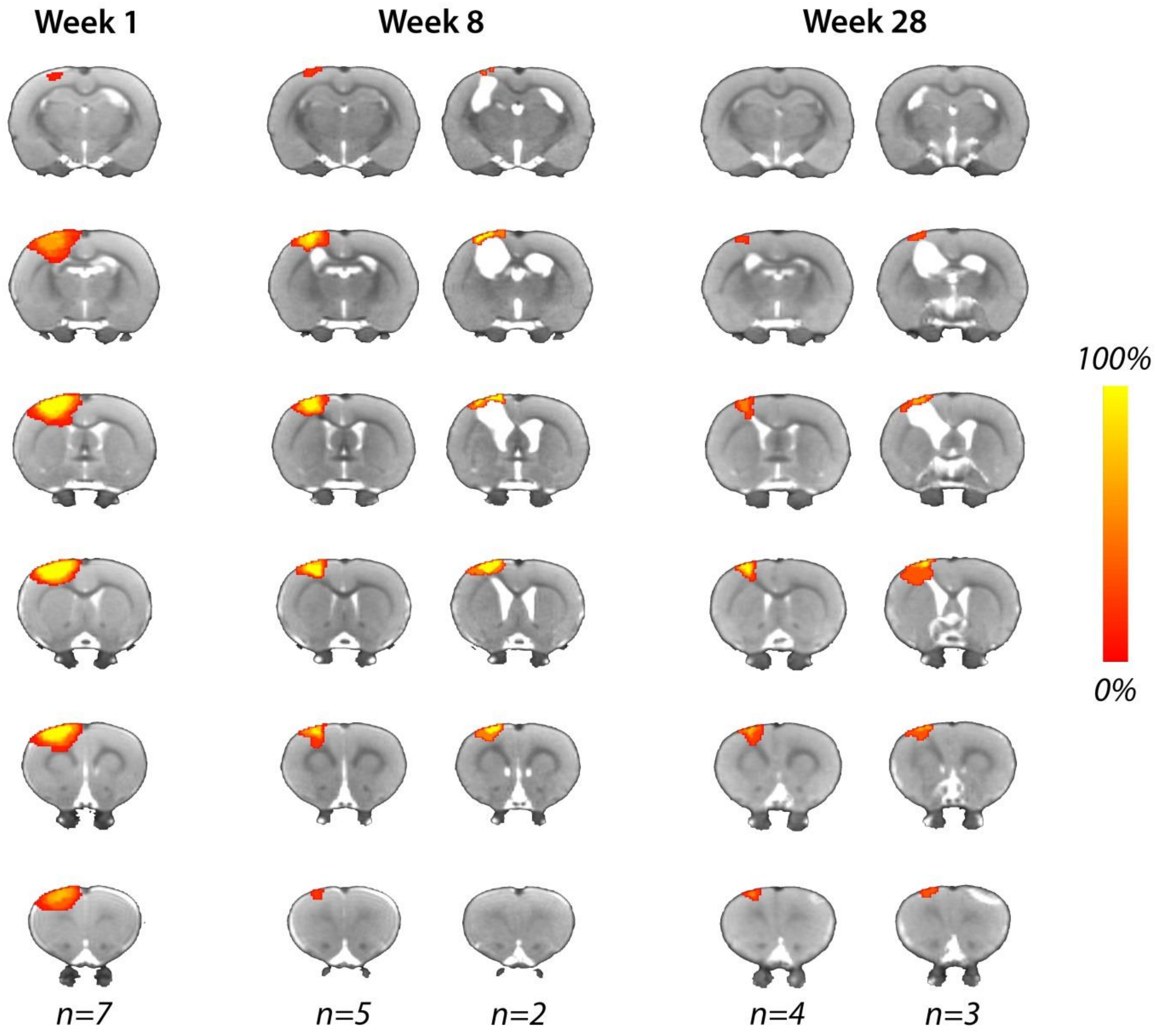

Suppl. Figure 3. Lesion incidence maps overlaid on anatomical rat brain images at 1, 8 and 28 weeks post-stroke. At 8 and 28 weeks post-stroke, ipsilateral ventricles were substantially enlarged in some animals. For illustration purposes, maps are shown from animals with minor (left columns) and severe ventricular enlargement (right columns) at 8 and 28 weeks after stroke.

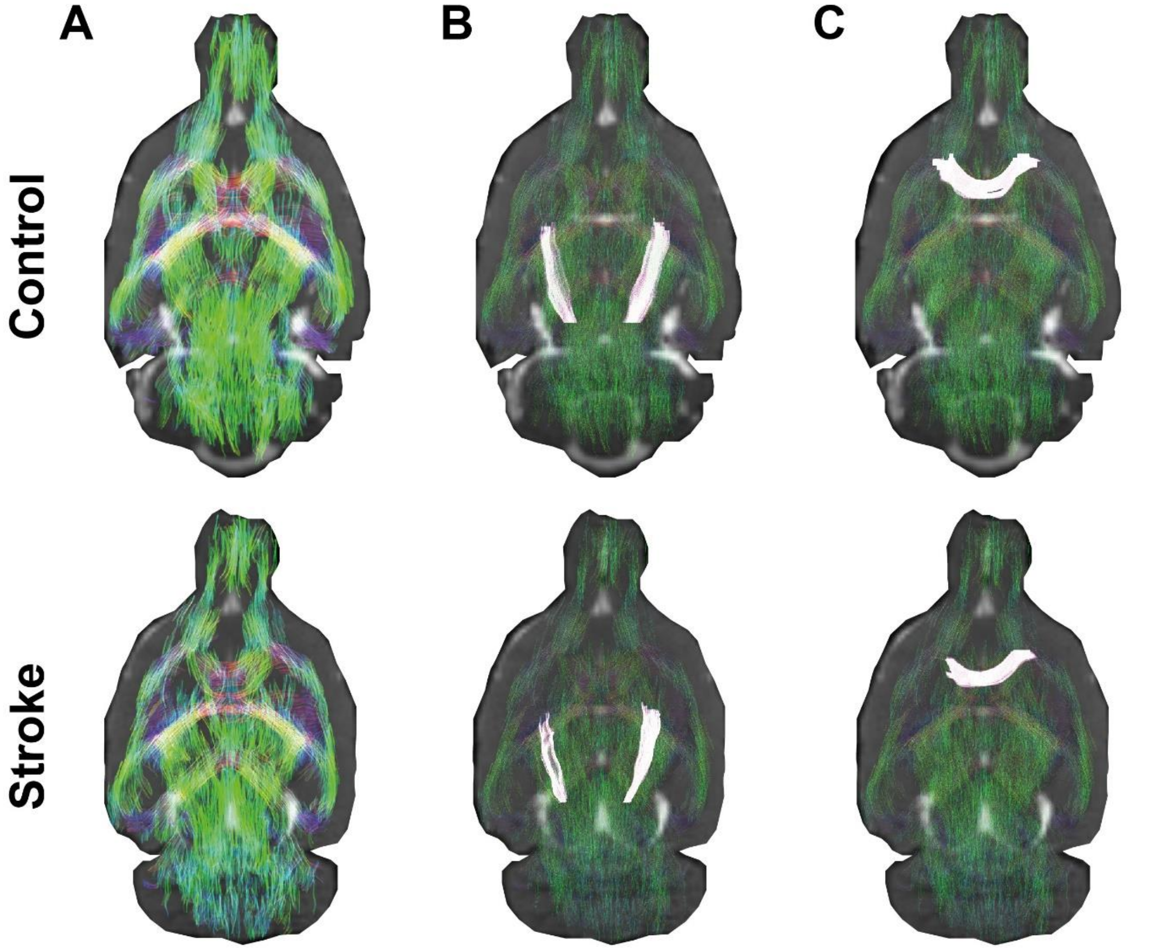

Suppl. Figure 4. Diffusion MRI-based tractography and subsequent segmentations. (A) Whole-brain tractograms and **(B)** representative segmentations (*white*) of the bilateral CST and **(C)** transcallosal tracts of a control rat (*top*) and stroke rat (*bottom*) projected on an anatomical image of the rat brain (sagittal plane).

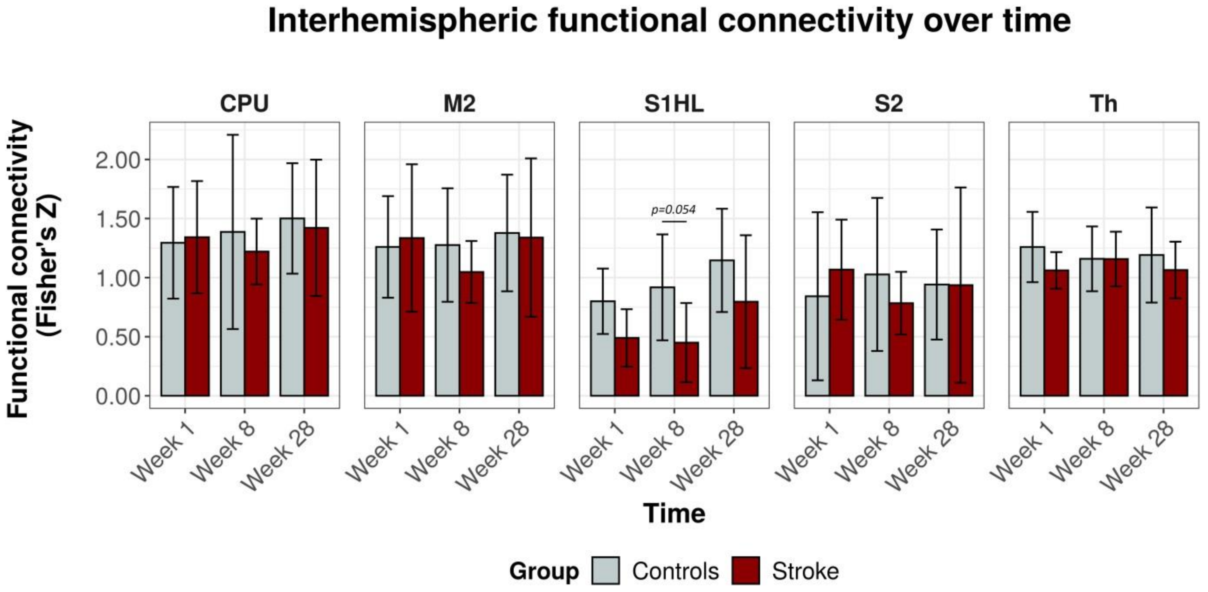

Suppl. Figure 5. Interhemispheric functional connectivity over time for different sensorimotor regions. Functional connectivity as mean (±SD) Fisher's Z for the caudate putamen (CPU), secondary motor cortex (M2), hindlimb regions of the primary somatosensory cortex (S1HL), secondary somatosensory cortex (S2) and thalamus (Th) at 1 week, 8 weeks and 28 weeks for both controls (*grey*) and stroke rats (*red*).

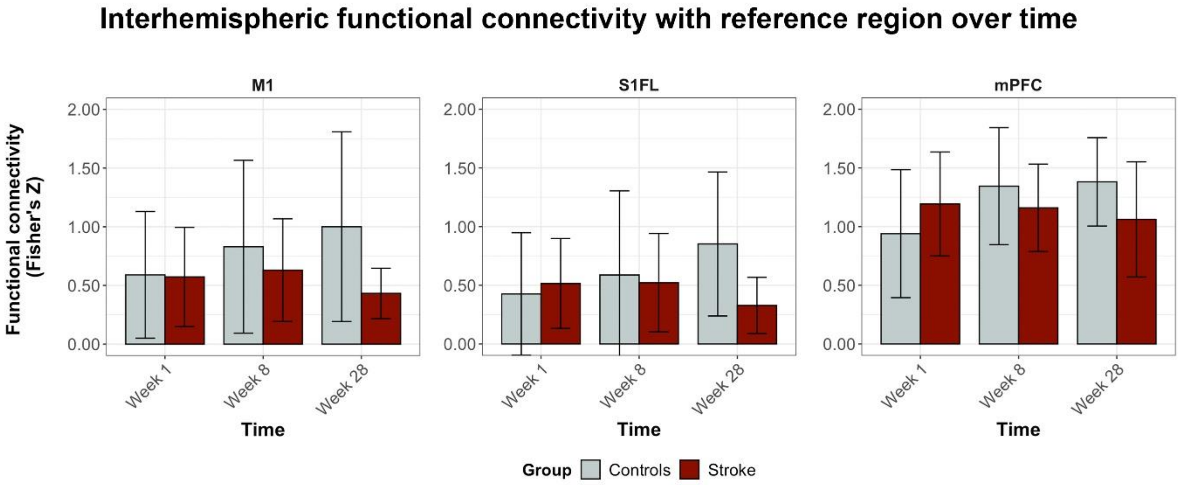

Suppl. Figure 6. Interhemispheric functional connectivity of the ipsilesional medial prefrontal cortex (mPFC) (a reference region in the default mode network) with the contralesional primary motor cortex (M1), the contralesional forelimb region of the primary somatosensory cortex (S1FL) and the contralesional mPFC, at different time points. Functional connectivity as mean (±SD) Fisher's Z at 1 week, 8 weeks and 28 weeks for both controls (*grey*) and stroke rats (*red*).

Tables

Suppl. Table 1: Number of reconstructed streamlines from diffusion tensor imaging

**Group**	**CC (mean ± SD)**	**CST left (mean ± SD)**	**CST right (mean ± SD)**
Control – week 1	1217 ± 886	1546 ± 671	1696 ± 1172
Stroke – week 1	1266 ± 674	1760 ± 751	610 ± 432
Control – week 8	1735 ± 909	2357 ± 1260	2365 ± 912
Stroke – week 8	795 ± 559	1836 ± 843	1086 ± 441
Control – week 28	1638 ± 1053	2058 ± 646	1721 ± 495
Stroke – week 28	556 ± 262	1921 ± 763	836 ± 394

CC: corpus callosum; CST: corticospinal tract.

References

1. Jenkinson M, Bannister P, Brady M, Smith S. Improved optimization for the robust and accurate linear registration and motion correction of brain images. *Neuroimage*. 2002;17(2):825-841. doi:10.1016/S1053-8119(02)91132-8.
2. Jenkinson M, Smith S. A global optimisation method for robust affine registration of brain images. *Med Image Anal*. 2001;5(2):143-156. doi:10.1016/S1361-8415(01)00036-6.
3. Andersson JLR, Jenkinson M, Smith S. *Non-Linear Registration, Aka Spatial Normalisation. FMRIB Technial Report TR07JA2.*; 2007.
4. Paxinos G, Watson W. *The Rat Brain in Stereotaxic Coordinates 5th Edition*. Elsevier Academic Press, Amsterdam; 2005.
5. Majka P, Kublik E, Furga G, Wójcik DK. Common atlas format and 3D brain atlas reconstructor: Infrastructure for Constructing 3D brain atlases. *Neuroinformatics*. 2012;10(2):181-197. doi:10.1007/s12021-011-9138-6
6. Hua Y, Schallert T, Keep RF, Wu J, Hoff JT, Xi G. Behavioral tests after intracerebral hemorrhage in the rat. *Stroke*. 2002;33(10):2478-2484. doi:10.1161/01.STR.0000032302.91894.0F
